# Diabetes mellitus early warning and factor analysis using ensemble Bayesian networks with SMOTE-ENN and Boruta

**DOI:** 10.1038/s41598-023-40036-5

**Published:** 2023-08-05

**Authors:** Xuchun Wang, Jiahui Ren, Hao Ren, Wenzhu Song, Yuchao Qiao, Ying Zhao, Liqin Linghu, Yu Cui, Zhiyang Zhao, Limin Chen, Lixia Qiu

**Affiliations:** 1 Department of Health Statistics, School of Public Health, Shanxi Medical University, Taiyuan, Shanxi China; 2 Shanxi Centre for Disease Control and Prevention, Taiyuan, 030012 Shanxi China; 3 Shanxi Provincial People’s Hospital, Taiyuan, Shanxi China

**Keywords:** Diseases, Medical research, Risk factors

## Abstract

Diabetes mellitus (DM) has become the third chronic non-infectious disease affecting patients after tumor, cardiovascular and cerebrovascular diseases, becoming one of the major public health issues worldwide. Detection of early warning risk factors for DM is key to the prevention of DM, which has been the focus of some previous studies. Therefore, from the perspective of residents' self-management and prevention, this study constructed Bayesian networks (BNs) combining feature screening and multiple resampling techniques for DM monitoring data with a class imbalance in Shanxi Province, China, to detect risk factors in chronic disease monitoring programs and predict the risk of DM. First, univariate analysis and Boruta feature selection algorithm were employed to conduct the preliminary screening of all included risk factors. Then, three resampling techniques, SMOTE, Borderline-SMOTE (BL-SMOTE) and SMOTE-ENN, were adopted to deal with data imbalance. Finally, BNs developed by three algorithms (Tabu, Hill-climbing and MMHC) were constructed using the processed data to find the warning factors that strongly correlate with DM. The results showed that the accuracy of DM classification is significantly improved by the BNs constructed by processed data. In particular, the BNs combined with the SMOTE-ENN resampling improved the most, and the BNs constructed by the Tabu algorithm obtained the best classification performance compared with the hill-climbing and MMHC algorithms. The best-performing joint Boruta-SMOTE-ENN-Tabu model showed that the risk factors of DM included family history, age, central obesity, hyperlipidemia, salt reduction, occupation, heart rate, and BMI.

## Introduction

Diabetes mellitus (DM) represents a metabolic disorder syndrome, which is characterized by abnormal elevation of blood glucose caused by a variety of factors^1,2^. According to statistics, the global prevalence of DM stood at 8.8% in 2017, with 425 million adults (aged 20–79 years) affected. In particular, the prevalence of DM in China reached 9.7%, and the number of people with DM reached 114 million, ranking first worldwide^3^. With an ageing population and people's improved living standards, the prevalence of DM is increasing annually. Therefore, the prevention and control of DM and its complications are of great significance^4^.

For high-risk groups, it is very important to detect risk factors, which represent the strong contributing factors of the outcome variable, having been wildly used in medical informatics for prediction and decision support^5,6^. If the risk factors detected are controlled, they will play a vital role in disease prevention. Therefore, the analysis of related factors and risk reasoning of DM could provide targeted prevention and control measures to prevent the occurrence and development of DM, so as to achieve better life quality and health.

Based on clinical data, many studies have employed various statistical methods and machine learning (ML) algorithms to build risk prediction models of DM. Among them, the most widely used methods represent Logit^7–9^ and Probit^10–12^. However, traditional statistical methods have several strict prerequisites for application, which are often not applicable to complex clinical data on DM. In addition, the predictive performance of traditional models is inferior to that of advanced ML algorithms in many medical studies. For example, in a study conducted by Yang et al.^13^, three models, weighted random forest, weighted SVM and logistic regression (LR), were applied to clinical data of liver cirrhosis with hepatic encephalopathy, and the results showed that the first two ML models enjoyed better prediction accuracy. Dinh et al.^14^ employed various ML algorithms for risk prediction of diabetes and cardiovascular disease, and the results also indicated that the XGBoost model and ensemble model showed the best prediction performance and outperformed the traditional LR model.

Bayesian networks (BNs), a classical machine learning method, have shown good predictive performance in many studies^15,16^, compared to traditional statistical methods (LR, MLR), as well as some advanced ML algorithms (RF, SVM, CNN). BNs are an uncertainty inference framework, which can directly describe the probabilistic structure of multivariate data by constructing a directed acyclic graph (DAG) to show the underlying relationships between variables and using conditional probability distribution tables (CPT) reflecting the strength of correlation^17,18^, so as to better present the complex network relationships between predictors and the outcome variable and reveal the direct and indirect relationships between influencing factors. This makes BNs fit for large-scale data mining in clinical settings^19,20^. For this purpose, the BNs was applied to establish a prediction model for inferring the risk of DM, and to portray the intrinsic relationships between DM and its related factors.

However, in a majority of clinical research, the number of patients is far less than that of the normal population, so there is often a class imbalance in the data. As mentioned earlier, although the prevalence of DM in the world is increasing annually, the prevalence is merely about 10%, and the number of the normal population is about 9 times that of those with DM. Therefore, there is a serious class imbalance in the DM data, which is undoubtedly a great challenge to the classification prediction algorithms. Because classification algorithms tend to improve the recognition of the majority class samples, causing a reduction in the recognition accuracy of the minority class samples^21^. In response to this issue, it is mainly solved from two aspects: algorithm level and data level^22^. The former is to add cost-sensitive analysis into some algorithms, in which the classes involved in the classification task are allocated different misclassification costs^23^. However, how to determine the best misclassification cost value for each class is a huge project^24^. The methods based on data level include resampling methods and case–control methods. Among them, resampling methods have been gradually applied in processing unbalanced data, due to their simplicity and ease of implementation^25–27^. However, some scholars believe that resampling may result in overfitting. Obviously, this issue has little impact on our study, as our focus is to detect factors significantly associated with DM, and the overfitting problem could not alter the actual relationships between dependent (DM) and independent variables (DM-related factors)^28^. Therefore, we choose the resampling approach to balance the data and then run the BNs models to analyze the DM data. Given that the SMOTE-ENN combination resampling method in resampling techniques combines the benefits of the SMOTE method and the Edited Nearest Neighbor (ENN) method, it first uses SMOTE to generate new samples to address data imbalance and then uses ENN to remove any examples whose class label differs from at least two of their three nearest neighbors. This approach effectively handles imbalanced data and eliminates noise. It has proven enhanced classification prediction performance when used in conjunction with classification algorithms in various studies and is regarded as a potential strategy for dealing with imbalanced class data^29,30^.

Furthermore, considering the non-variable screening model, not only has poor reproducibility in various medical settings, but also incurs huge computational costs in operation and post-maintenance, so it is not suitable for clinical practice. Thus, we adopted Boruta feature screening to further screen the factors, which has been widely used in clinical medicine by iterative processing to deal with random fluctuations in random forest importance scores and the interaction between factors, screening out the most important predictors for DM^31–33^. This method also has been widely used in DM research for feature selection. For instance, Zhou et al. combined the Boruta feature selection method with various machine learning algorithms^34^. Mengting Li et al. also applied the Boruta method for variable selection in their study on the development and assessment of novel machine learning models to predict medication non-adherence risks in type 2 diabetics^35^. Hahn et al. utilized the Boruta method and LR/RF classification models for predicting type 2 diabetes^36^. In these studies, the classification models that incorporated Boruta for feature selection demonstrated superior performance in diabetes classification prediction. These findings highlight the widespread recognition and effectiveness of Boruta in feature selection.

Considering the advantages of Boruta, SMOTE-ENN and BNs in their respective fields, our paper primarily aims at combining the approaches of Boruta, SMOTE-ENN and BNs to explore the relationships between demographics, lifestyle, physical condition and DM, and to detect the warning factors for DM, which will contribute to the prevention and control of DM, as well as the development of community work, facilitating the monitoring of DM patients and health management.

## Methods

### Study participants

The subjects came from 8 surveillance sites included in the adult chronic diseases and nutrition surveillance in Shanxi Province in 2018. A total of 4886 subjects were chosen using multi-stage stratified random cluster sampling. This study was approved by the Chinese Chronic Disease Center Ethics Committee (No. 201819). Informed consent was signed by all participants or their agents in this study. All experiments were conducted in accordance with the relevant guidelines and regulations.

#### Inclusion criteria

(1) Age ≥ 18 years old;

(2) Permanent residents who have resided in Shanxi Province for more than 6 months before the survey.

#### Exclusion criteria

(1) Women during pregnancy;

(2) People with cognitive impairment;

(3) People suffering from serious diseases or disabilities that may affect the investigation;

(4) People who refuse to participate in the project;

(5) Residents who lived in functional areas, such as military or student dormitories, sheds, nursing homes, and so on.

#### Sampling method

This study adopted the method of multi-stage stratified random cluster sampling, which included the following four stages:

Stage 1: 3 randomly selected townships (streets/regiments) in each of the 8 monitoring sites in Shanxi Province using the systematic sampling method ranked by population size;

Stage 2: 2 administrative villages (neighborhood committees/companies) were randomly selected from each township (street/regiment) using the systematic sample ranked by population size;

Stage 3: Within each sampled administrative village (neighborhood committee/company), residential households were divided into villagers/resident groups on a scale of at least 60 households and one village/resident group was selected by the simple random sampling method;

If less than 100 permanent residents aged 18 and above have completed the individual survey among the 45 sampled households, the corresponding survey households should be selected from the remaining households in that village/resident group for supplementary surveys;

Stage 4: About 45 households in each sampled village/resident group were selected to survey the residents aged 18 and above in the surveyed households. If less than 100 permanent residents aged 18 and above had completed the individual survey among the 45 sampled households, the corresponding survey households should be selected from the remaining households in that village/resident group for additional surveys. Each monitoring site should survey at least 600 permanent residents aged 18 and above.

### Survey methods

Before conducting the on-site survey, the identification information of respondents was confirmed, and all the subjects voluntarily signed the informed consent. After the subjects were formally enrolled, they underwent the questionnaire, physical examination, and laboratory examination. The Chinese Adult Chronic Disease and Nutrition Surveillance Questionnaire (2018) were used to survey by face-to-face inquiry. The contents included: basic information (gender, age, occupation, educational level, marital status), behavioral lifestyle (smoking, drinking, physical activity, dietary habits) and health status (the incidence and control of various chronic diseases). After confirming that the subjects met the requirements for physical examination, the investigators conducted a physical examination of the subjects using standard methods as required by the study protocol, including weight, height, blood pressure and waist circumference. Laboratory test indicators included blood lipids, blood sugar, glycosylated hemoglobin, etc. The blood glucose test samples should be stored in the refrigerator at 2 ~ 8℃, then sent to the local designated laboratory within 48 h; Other samples should be stored at a low temperature between − 60℃ and − 80℃. In the absence of ultra-low temperature storage facilities, the samples shall be stored below − 20℃ and sent to the nationally designated medical inspection agency to be determined in one month.

### Definitions

(1) The diagnostic criteria for DM were defined as fasting plasma glucose (FPG) ≥ 7.0 mmol/L, 2-h postprandial glucose (2hPG) ≥ 11.1 mmol/L or already diagnosed with DM^37^; (2) Hypertension was defined as systolic blood pressure(SBP) ≥ 140 mmHg and/or diastolic blood pressure (DBP) ≥ 90 mmHg, and included patients with a prior history of hypertension and those currently taking antihypertensive medications^38^; (3) According to the Guidelines for Prevention and Treatment of Dyslipidemia in Chinese Adults (revised edition 2016), total cholesterol (CHOL) ≥ 5.18 mmol/L and (or) triglyceride (TG) ≥ 1.70 mmol/L and (or) low-density lipoprotein cholesterol (LDL-C) ≥ 3.37 mmol/L and (or) high-density lipoprotein cholesterol (HDL-C) ≤ 1.04 mmol/L, and the person that has a history of hyperlipidemia before is defined to have hyperlipidemia^39^. (4) Body mass index (BMI): Body weight was categorized as underweight (BMI < 18.5 kg/m^2^), normal weight (BMI: 18.5 kg/m^2^ ~ 24 kg/m^2^), overweight (BMI: 24 kg/m^2^ ~ 28 kg/m^2^), and obesity (BMI ≥ 28 kg/m^2^)^40^; (5) Central obesity was defined as male waist circumference ≥ 85 cm, female waist circumference ≥ 80 cm^40^. Physical activity was divided into insufficient physical activity and sufficient physical activity, and the classification standard was whether the activity of moderate-intensity or above exceeded 150 min per week. The characteristics of other underlying diseases were obtained by questionnaire inquiry.

### Data pre-processing

Firstly, the quantitative data (such as age, heart rate and lipid index) were discretized reasonably. Then, the samples with too much missing information or could not be determined whether they had DM were deleted. For the samples with little missing information (Delete loss rate < 30%), mode-based imputation was employed to fill in the missing values.

### Feature selection

Boruta, proposed by Kursa et al.^41^, is a feature selection algorithm based on the random forest (RF) classifier. It deals with the random fluctuation of the importance scores of RF and the interaction among independent variables through an iterative process. This method is mainly divided into five steps: (1) Create shadow variables and a new feature matrix N: all variables are shuffled to produce new variables, called "shadow variables", forming a feature matrix S and are stitched together with the original data (named “R”) to form a new matrix N = [R, S]; (2) The model based on the new feature matrix N training can output variable importance(VIM) score; (3) Calculate the Z-score values of original variables and shadow variables: Z-Score = average(VIM)/SD(VIM); (4) Determine whether the original variable is important: take the largest Z-score among shadow variables, mark it as MZSA (Maximum Z Score among shadow attributes), and perform a two-sided test on whether the Z-score of each original variable is equal to MZSA. Variables with a larger Z-score are labelled as "important variables", and variables with a smaller Z-Score are defined as "non-important variables"; (5) Delete the original variables marked as "non-important" and all shadow variables; (6) Repeat the above process until all variables are marked as "important" or reach the preset RF construction times in a certain cycle.

### Resampling techniques

#### Synthetic Minority Oversampling Technique (SMOTE)

Proposed by Chawla in 2002, the main idea of SMOTE is to synthesize new samples based on linear interpolation^42^. It is assumed that the minority class samples (DM patients) in the original data is *i*, and the feature vector of DM patients is denoted as *X*_*i*_, *i* ∈ (1, 2, …, *k*). Find the *k* nearest neighbor samples from all *T* samples of DM patients and denote them as *X*_*i(near)*_, *near* ∈ (1, 2, …, *k*). Repeating the above steps to find all the nearest neighbor samples, and then randomly select 1 sample from *k* nearest neighbor samples, denoted as *X*_*inn*_, and then generate a random number, i.e., *ζ*_1_ within the range of 0 to 1, so as to synthesize a new sample according to the formula (1). If the above steps are repeated for N times, N new samples can be synthesized. If the above steps are carried out for all DM samples, NT new samples can be synthesized, so as to achieve minority class samples expansion.

1 $$\begin{array}{*{20}c} {X_{i1} = X_{i} + \zeta_{1} \left( {X_{inn} - X_{i} } \right)} \\ \end{array}$$

#### Borderline-SMOTE (BL-SMOTE)

Borderline-SMOTE is an improved oversampling algorithm based on SMOTE^43^. Its main idea is to first divide the minority class samples (DM patients) into three classes of samples: safe, border and noisy according to certain rules, and to perform nearest-neighbor linear interpolation only for the border samples (as in SMOTE) so that the synthesized minority class samples are more reasonably distributed.

#### SMOTE-ENN

Proposed by Batista and Prati^44^, SMOTE-ENN represents a hybrid sampling method. Combining the merits of SMOTE^42^ and edited nearest neighbor (ENN)^45,46^, the algorithm can effectively process imbalanced data and remove noise. SMOTE is employed to deal with data imbalance. ENN is used as a data cleaning method ,which can delete any example whose class label differs from the class of at least two of its three nearest neighbors^47^. Since some majority class examples may invade the minority class space and vice versa, SMOTE-ENN reduces the possibility of overfitting introduced by synthetic examples^44^.

### Bayesian network

Bayesian networks (BNs), also known as causal networks or belief networks, were proposed by Pearl Judea in 1988^48^. Based on probability theory, BN represents a probability graph model that uses a DAG to represent the conditional dependence relationship between a group of random variables and is suitable for analyzing uncertainties and probabilistic events^49^. BNs combine the probabilistic method with the graph structure organically and integrate the prior knowledge and the existing statistical data to deduce and predict unknown events in the form of probability. It not only has a solid mathematical foundation, but also provides a simple topological structure to describe the intrinsic interdependencies of the data intuitively.

#### Bayesian network structure

Suppose there is a set of random variables $X = \left\{ {X_{1} ,X_{2} ,X_{3} , \ldots ,X_{n} } \right\}$ and $P\left( {X_{1} ,X_{2} ,X_{3} , \ldots ,X_{n} } \right)$ denotes the joint probability distribution of the variables in the set, then the BN can be presented as *B* = (*G*, *P*)^50^. *G* represents a directed acyclic graph, including nodes (random variables) and directed line segments. In BNs, if there is a directed edge pointing from node *X*_1_ to node *X*_2_, then the node *X*_1_ represents the parent node of *X*_2_; in other words, a node *X*_2_ is the child node of *X*_1_. Nodes that have no parents are called root nodes, the rest are called non-root nodes, and nodes that have both parents and children are called intermediate nodes. Directed edges represent the dependence between random variables, which can also be called the causal relationship of conditional dependence. The strength of the dependence is expressed by the conditional probability between some node and its parent node, and the node without the parent node is expressed by prior probability. It should be noted that a directed loop cannot be formed between nodes of BNs. BNs can also be understood from qualitative and quantitative aspects. The dependent or independent relationship between variable nodes is described qualitatively utilizing a directed acyclic graph, and a conditional probability distribution table is used to quantitatively describe the degree of dependency between variable nodes. Semantically, it is the form of decomposing the joint probability distribution.

#### Tabu algorithm

The Tabu search^51^ algorithm is based on a feasible initial solution, searches for the optimal solution of the nearest neighbor of the initial solution through local neighborhood movement, stores the obtained local optimal solution in the tabu table, and then takes the local optimal solution as the initial solution and continues to move to the neighborhood. Repeat the above process, reject the new optimal solution if it already exists in the taboo list, and remove some taboo solutions with flouting criteria, to avoid circuitous search, and ensure that diversification is effectively explored and ultimately optimized globally. By constructing a taboo list and related criteria, the Tabu Search algorithm can well overcome the problem of falling into local optimization.

#### Hill-Climbing Algorithm

The Hill-Climbing Algorithm^52^ aims to identify the model with the highest score. Initially, an unbounded model is often chosen as the starting point. During each step of the search, the current model is locally modified using three search operators: edge addition, edge subtraction, and transition edge. This process generates a series of candidate models. The score of each candidate model is then calculated and compared to determine the optimal candidate model concerning the current model. If the optimal candidate model has a higher score, the search continues using it as the new current model. Conversely, if the optimal candidate model has a lower score, the search terminates, and the current model is returned. However, due to the initial network selection, the Hill-Climbing Algorithm is susceptible to getting trapped in local optima and fails to discover the globally optimal network.

#### Max–Min Hill-Climbing (MMHC)

The MMHC algorithm combines the Constraint-based (CB) algorithm and the Scoring and searching (SS) algorithm, which consists of two stages: (1) Utilizing the Max–Min Parents and Children algorithm (MMPC) to determine the candidate parents and children (CPC) set for each variable node and construct the framework of BNs structure, and (2) performing a score-based search using the Hill-Climbing Algorithm to determine the edges and directions of the BNs structure^53^. MMPC employs a two-stage scheme to obtain the CPC for each node from the given dataset: in the first stage, variables are sequentially input into the CPC of the target node T using the Max–Min heuristic function until any remaining node becomes independent of T, and the first stage stops. In the second stage, the MMPC algorithm removes variables mistakenly included in the CPC during the previous stage; if there exists a variable X from CPC of T make the $Ind\left( {X,T|Y} \right)$ such that holds, X is removed from the CPC. The Hill-Climbing Algorithm starts with an initial model without any edges and performs operations such as adding edges, removing edges, and reversing edges to locally modify the current initial model, generating multiple candidate models. Subsequently, the scores of each candidate model are computed and compared to identify the network structure model with the highest score^52^.

In this paper, the maximum likelihood estimation method was applied for parameter learning.

### Evaluation index

In this study, we used several standard performance indicators: namely, Accuracy, Specificity, Sensitivity, NPV, PPV, MCC, G-mean, and the area under the receiver-operating characteristic curve (AUC) to evaluate the classification performance of the BNs. These matrices are computed by a binary confusion matrix.

When the DM subject is classified as DM, the predicted output is defined as True Positive (TP), and when the healthy person is classified as healthy, True Negative (TN). False Positive (FP) when a healthy subject is treated as a DM and likewise False Negative (FN) when a patient with DM is regarded as a healthy subject. Then, the performance evaluation matrix is calculated using the four confusing matrices.

$$\begin{aligned} {\text{Accuracy}} & = \frac{{\left( {TN + TP} \right)}}{{\left( {TP + TN + FP + FN} \right)}} \times 100\% \\ {\text{Specificity}} & = \frac{TN}{{\left( {TN + FP} \right)}} \times 100\% \\ {\text{Sensitivity}} & = \frac{TP}{{\left( {TP + FN} \right)}} \times 100\% \\ {\text{PPV}} & = \frac{TP}{{TP + FP}} \times 100\% \\ {\text{NPV}} & = \frac{TN}{{TN + FN}} \times 100\% \\ {\text{MCC}} & = \frac{TP \times TN - FP \times FN}{{\sqrt {\left( {TP + FP} \right)\left( {TP + FN} \right)\left( {TN + FP} \right)\left( {TN + FN} \right)} }} \times 100\% \\ {\text{G - mean}} & = \sqrt {\frac{TP}{{TP + FN}} \times \frac{TN}{{TN + FP}}} \times 100\% \\ \end{aligned}$$

### Statistical analysis

The data were statistically described using IBM SPSS Version 26 (IBM Corp., Armonk, NY, USA).

Significance for all statistical tests was a priori at *P* < 0.1 and all *P* values were two-tailed; Python (version 3.9.7) was employed to carry out SMOTE, Borderline-SMOTE and SMOTE-ENN resampling. R studio 4.0.5 (R Development Core Team) was adopted to implement the feature dimension reduction, i.e., Boruta. The BNs structure was constructed using the “bnlearn” packages in R Studio 4.0.5 (R Development Core Team), and the maximum likelihood method was used for parameter learning. The graphs in this article were drawn in Excel, Netica (Norsys Software Corp., Vancouver, BC, Canada) and R Studio 4.0.5 (R Development Core Team).

### Ethics approval

This study has been approved by the China Chronic Disease Center Ethics Committee (No. 201819). All study participants or their agents signed the informed consent. All experiments were carried out under relevant guidelines and regulations.

## Results

### Characteristics of the study population

Of the 4886 initial participants, we excluded 95 respondents with incomplete data. Eventually, a total of 4791 participants (2153 males and 2638 females) were enrolled in this study; 3193 (66.7%) participants were from rural regions and 1598 (33.3%) from urban areas. The median age of the total population was 55.4 years, ranging from 18 to 88 years.

**Figure 1:**
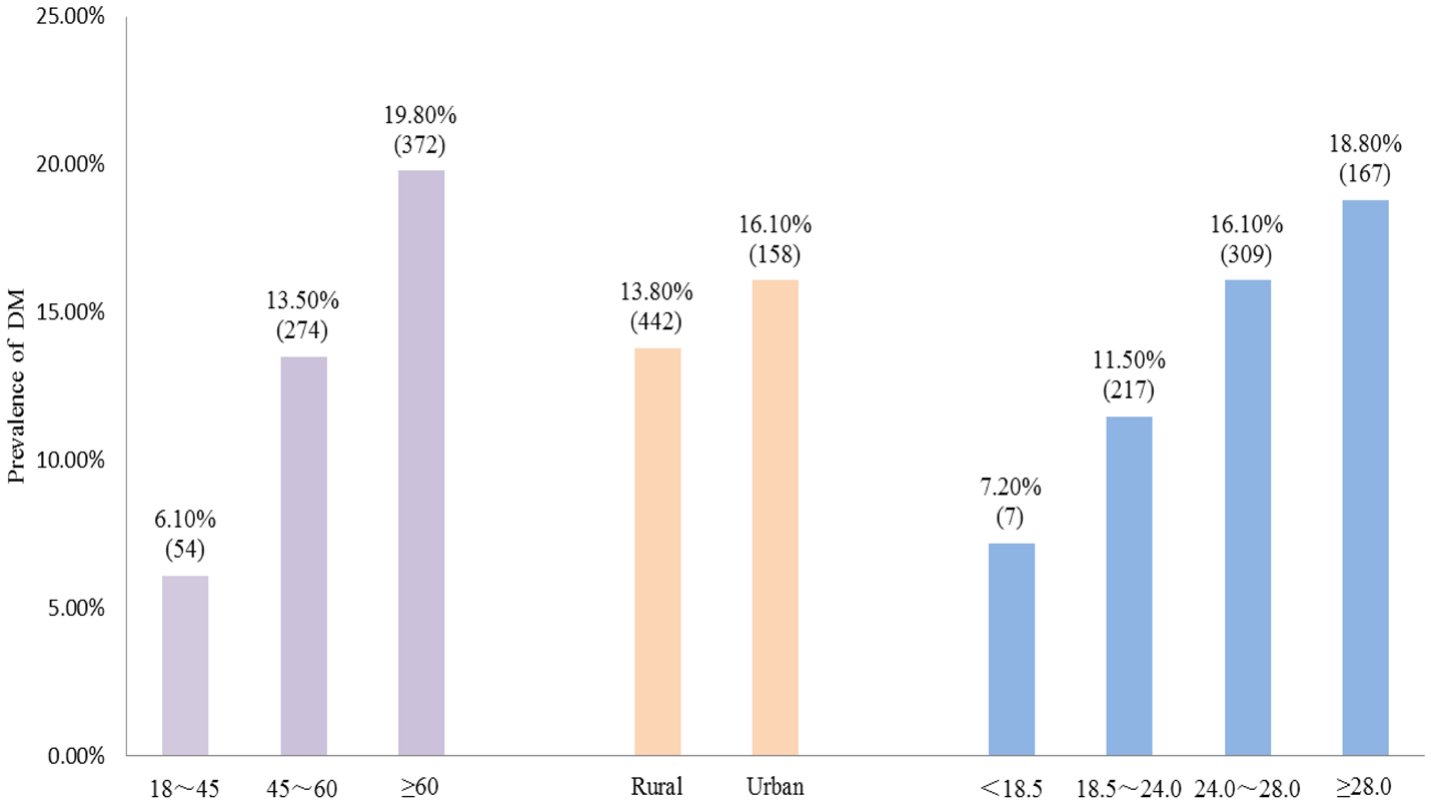
The prevalence of DM in different regions, ages and BMI.

There were 700 patients with DM and the prevalence was 14.6%. Moreover, as age increased, the prevalence increased, rising from 6.1 to 19.8%; the prevalence of DM was slightly higher in urban areas than in rural areas; the higher the BMI, the higher the prevalence of DM and the highest prevalence reached 18.8% in the obese group (as shown in Fig. 1).

### Univariate analysis

The distribution of DM patients among different factors and univariate analysis results were presented in Table S1–3. The univariate factor analysis was realized using the chi-square test, and the test level *α* was set at 0.10. The findings revealed that the prevalence of DM was significantly different across groups for 17 factors including age, educational level, marital status, region, occupation, drinking, physical activity, sleep duration, agrypnia, and mediation time (see Tables S1–3 for details on other components) (*P* < 0.1).

### Variable selection by Boruta

**Figure 2:**
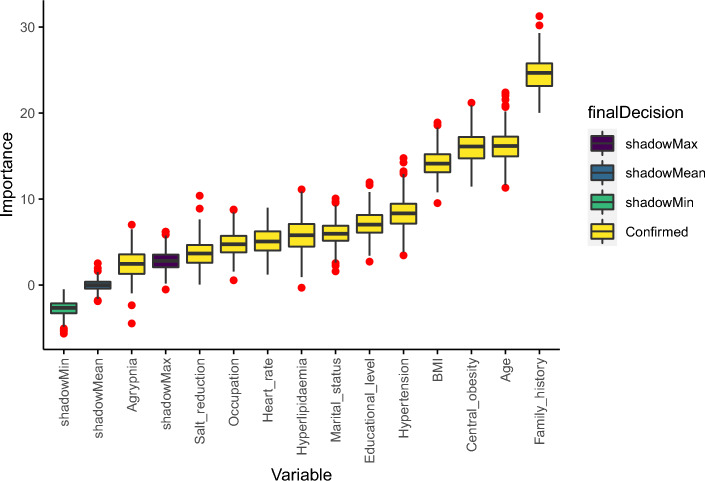
Variable selection using Boruta.

To raise the predictive performance of the model, the 17 variables mentioned above were selected for further feature screening using the Boruta method. This approach can capture all relevant characteristics for the classification in terms of importance. Figure 2 implies the importance of the y-axis of the analyzed attributes (x-axis).

To obtain the relevant variables, Boruta performed 100 iterations, and the selection results were summarized in Table 1. Out of 17 features, 4 were rejected, one was tentative, and 12 were confirmed. Additionally, the Norm Hits represented the number of the RF ran where this feature was more important than the shadow one.

**Table 1:** Feature selection results—confirmed attributes.

No	Attribute	Feature selection-Boruta norm hits
1	Family history	1.0000
2	Age	1.0000
3	Central obesity	1.0000
4	BMI	1.0000
5	Hypertension	1.0000
6	Educational level	0.9766
7	Marital status	0.9499
8	Hyperlipidemia	0.9398
9	Heart rate	0.8963
10	Occupation	0.8629
11	Salt reduction	0.6923
12	Agrypnia	0.4181

### Classification performance of BNs models based on different resampling techniques

**Figure 3:**
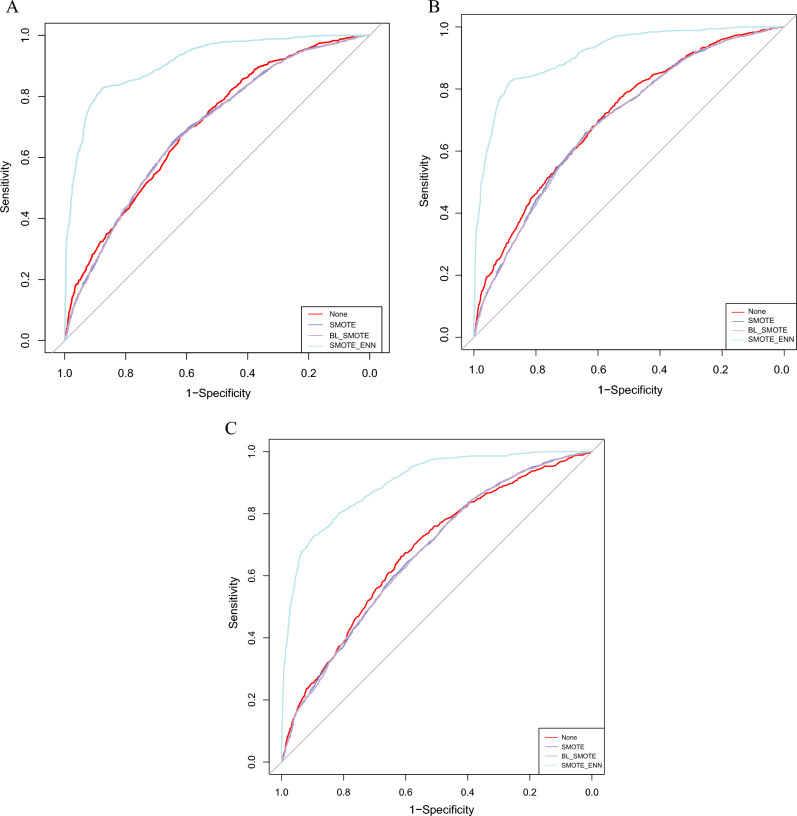
ROC curves of various BNs models under different resampling methods. (**A**) BNs models constructed by Tabu algorithm; (**B**) BNs models constructed by Hill-climbing algorithm; (**C**) BNs models constructed by MMHC algorithm.

To handle the imbalance in the original DM dataset, we employed three resampling techniques, SMOTE, BL-SMOTE and SMOTE-ENN for data balancing to obtain three new balanced datasets. Table 2 showed the distribution of respondents in the original unbalanced dataset and three balanced datasets. Twelve BNs network models were constructed based on these four datasets using Tabu, hill-climbing and MMHC hybrid algorithms, respectively, and the classification performance of each model is shown in Table 3 and Fig. 3. The results showed that the BNs enjoyed high accuracy, specificity and negative predictive value in the unbalanced original dataset, but were extremely poor at identifying diabetic patients (Sensitivity was merely 0.069, 0.067 and 0), which also suggests that it’s not reasonable to take accuracy alone as the criterion for evaluating classification models in extremely unbalanced datasets. However, the sensitivity of the BNs models constructed from the three new balanced datasets was significantly improved, especially the BNs combined with SMOTE-ENN, which scored higher in all the indicators and enjoyed the best classification performance. Only the specificity value was slightly lower than that of the BNs model based on the original dataset. This indicated that the application of resampling techniques to deal with data imbalance can improve the classification performance of models. This phenomenon was consistent across the three BNs algorithms, and the SMOTE-ENN-Tabu model had the best classification performance among the three BNs methods, with the highest accuracy (0.863), sensitivity (0.714), NPV (0.877), AUC (0.913), MCC (0.673), and G-mean (0.815). Therefore, subsequent analysis of diabetes-related factors and Bayesian network inference research was conducted using BNs models constructed based on the Tabu algorithm.

**Table 2:** Distribution of total participants and DM patients in the original and resampled datasets.

Dataset	N	DM	Non-DM
Original data	4791	700	4091
SMOTE	8182	4091	4091
BL-SMOTE	8182	4091	4091
SMOTE-ENN	3929	1232	2697

**Table 3:** Performance of BNs in different datasets.

Algorithms	Dataset	Accuracy	Sensitivity	Specificity	PPV	NPV	AUC	MCC	G-mean
Tabu	Original Data	0.854	0.069	0.989	0.511	0.861	0.695	0.146	0.260
	SMOTE	0.646	0.684	0.607	0.635	0.658	0.692	0.292	0.644
	BL-SMOTE	0.644	0.694	0.604	0.633	0.656	0.694	0.288	0.642
	SMOTE-ENN	0.863	0.714	0.931	0.825	0.877	0.913	0.673	0.815
Hill-climbing	Original Data	0.854	0.067	0.989	0.511	0.861	0.705	0.145	0.258
	SMOTE	0.646	0.687	0.605	0.635	0.659	0.694	0.292	0.645
	BL-SMOTE	0.645	0.685	0.605	0.634	0.658	0.692	0.291	0.644
	SMOTE-ENN	0.860	0.703	0.932	0.826	0.873	0.912	0.666	0.810
MMHC	Original Data	0.854	0.000	1.000	-	0.854	0.674	-	0.000
	SMOTE	0.619	0.733	0.504	0.597	0.654	0.668	0.244	0.608
	BL-SMOTE	0.623	0.739	0.507	0.600	0.660	0.670	0.253	0.612
	SMOTE-ENN	0.857	0.668	0.943	0.842	0.862	0.900	0.656	0.794

### Analysis of influencing factors of Diabetes Mellitus

Obviously, through the BNs, we can learn the direct dependence between diabetes and other warning factors, as well as the interdependence between these factors. However, since we are interested in the factors that contribute to the onset of DM, this paper focuses on exploring important predictors of DM rather than probabilistic dependence between them.

Accordingly, the factors directly related to DM in different BNs were illustrated in Table 4. BNs can find more predictors directly related to DM in the dataset processed using SMOTE-ENN. As expected, the issue of data imbalance may obscure the important relationship between DM and related factors, making it difficult for us to identify these important factors, and thus drawing incorrect conclusions. It means that appropriate data balancing techniques must be used to reduce such impacts. The BNs models combined with SMOTE, BL-SMOTE and SMOTE-ENN could classify and predict DM, suggesting that the warning factors they jointly found were strongly associated with DM, i.e., age, family history, hyperlipidemia, and central obesity.

**Table 4:** The warning factors directly associated with DM in different datasets.

	Original dataset			SMOTE-dataset		
Dependence relationship between DM and warning factors	DM	→	Family history	DM	→	Family history
	DM	→	Age	DM	→	Age
	–	–	–	DM	→	Central obesity
	DM	→	Hyperlipidemia	DM	→	Hyperlipidemia
	–	–	–	DM	→	Salt reduction
	–	–	–	–	–	–
	–	–	–	–	–	–
	–	–	–	–	–	–
	–	–	–	–	–	–
No. of dependence	3			5		
	BL-SMOTE-dataset			SMOTE-ENN-dataset		
Dependence relationship between DM and warning factors	DM	→	Family history	DM	→	Family history
	DM	→	Age	DM	→	Age
	DM	→	Central obesity	DM	→	Central obesity
	DM	→	Hyperlipidemia	DM	→	Hyperlipidemia
	DM	→	Salt reduction	DM	→	Salt reduction
	–	–	–	DM	→	Occupation
	–	–	–	DM	→	Heart rate
	–	–	–	DM	→	BMI
No. of dependence	5			7		

**Figure 4:**
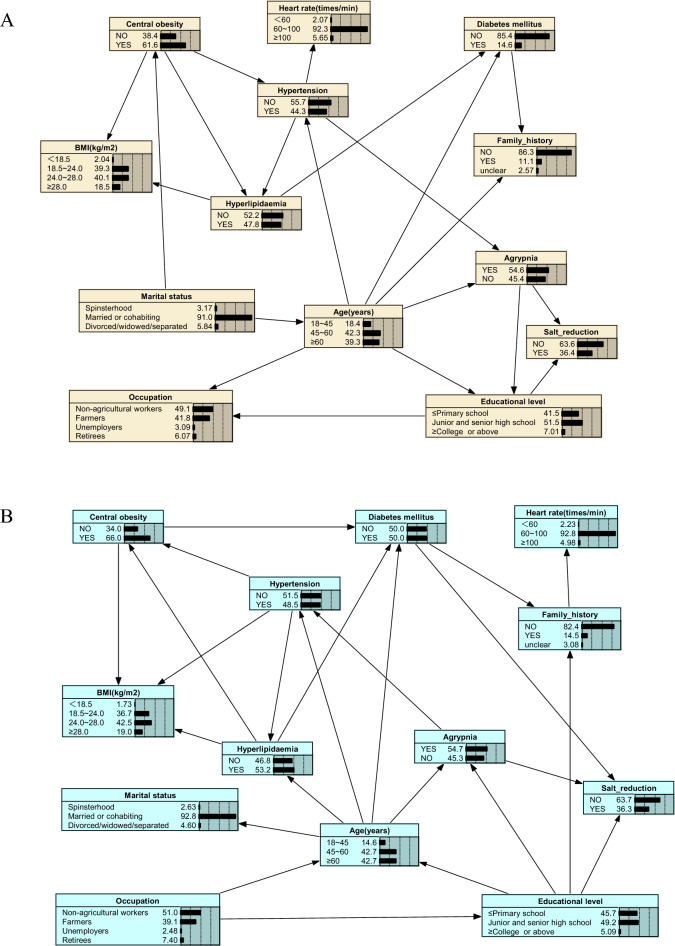

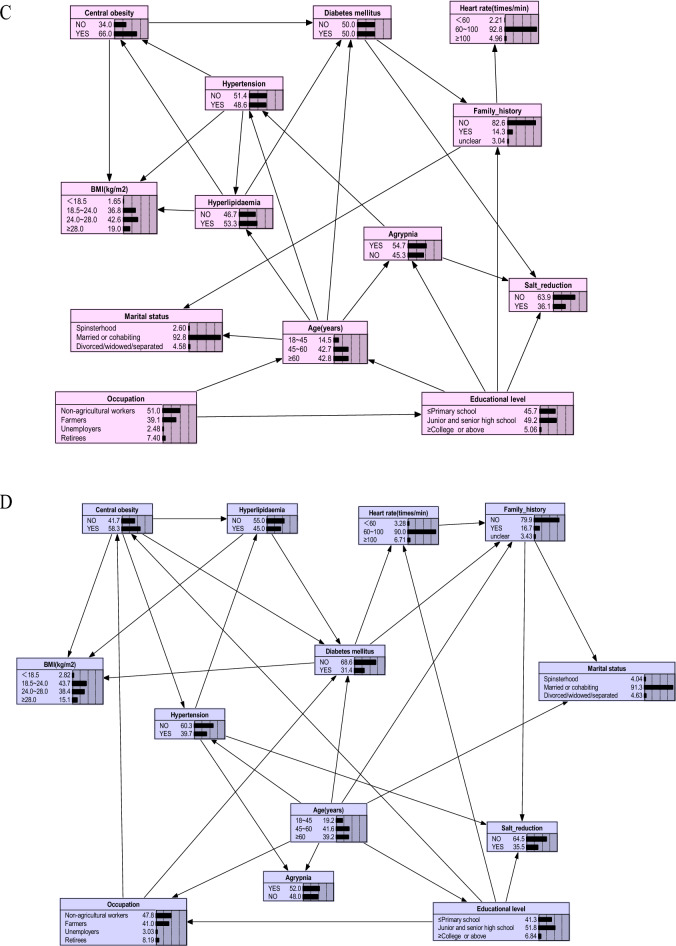
The Bayesian networks based on various datasets. (**A**) Original data-based Bayesian network graphs; (**B**) data-balanced Bayesian network graphs based on the SMOTE algorithm; (**C**) data-balanced Bayesian network graphs based on the BL_SMOTE algorithm; (**D**) data-balanced Bayesian network graphs based on the SMOTE-ENN algorithm. The figure was plotted by Netica (www.norsys.com).

Also, we vividly presented the complex network relationship between the factors found by BNs in the original dataset and three balanced datasets processed using different resampling methods in Fig. 4A–D. Based on Fig. 4, we have not only identified the direct influencing factors associated with the occurrence of DM, but also uncovered several indirect factors that influence diabetes occurrence. For instance, occupation impacts DM through central obesity. Previous studies mostly treated occupation and central obesity as independent factors for DM, whereas in this study, we found that occupation serves as both a direct influencing factor for DM and indirectly affects DM occurrence through central obesity. Given the challenges of intervening by modifying occupation for DM prevention, we can consider reducing the prevalence of central obesity among residents as a preventive measure. Like occupation, the educational level can also affect DM occurrence through central obesity (Fig. 4D). Agrypnia, through its influence on hypertension and dyslipidemia, contributes to the development of DM (Fig. 4B,C). This suggests that individuals with agrypnia are more prone to elevated blood pressure, and hypertension is often associated with abnormal lipid levels, thereby increasing the risk of DM. Effectively managing agrypnia may have a significant impact on the control of blood pressure, lipid levels, and blood glucose. The network connections among these factors highlight insights that are not attainable through traditional statistical learning methods or "black boxes" machine learning methods such as Random Forest and XGBoost.

### BN inference

To make the conclusions more accurate and reliable, we took the risk factors shared by the three BNs models (constructed combining SMOTE, BL-SMOTE and SMOTE-ENN approaches) as the real determinants of DM, including age, family history, hyperlipidemia and central obesity. BNs can infer the probability of an unknown node from the state of known nodes, so that it is possible to predict DM risk. Table 5 depicted the results of risk prediction for the four warning factors. For instance, if an individual is ≥ 60 years old, the probability of suffering from DM is 43.8%, namely *P*_*(DM|Age*≥*60)*_ = 43.8%, as shown in Table 5 (as shown in the Prob. of DM in SMOTE-ENN.BN.Tabu); similarly, if this person also owns a family history of DM, the probability of suffering DM will increase to 96.9%, namely *P*_*(DM|Age*≥*60, Family history)*_ = 96.9%.

**Table 5:** Risk reasoning for warning factors significantly associated with DM.

No. Var	Condition satisfied	Prob. of DM in BN.Tabu	Prob. of DM in SMOTE.BN.Tabu	Prob. of DM in BL-SMOTE.BN.Tabu	Prob. of DM in SMOTE-ENN.BN.Tabu
	P(DM)*	14.6	50.0	50.0	31.4
1	P(DM|Age ≥ 60)	19.9	62.6	57.2	43.8
2	P (DM|Family history = YES)	31.6	69.8	69.8	82.0
3	P (DM|Age ≥ 60, Family history = YES)	51.0	76.5	76.5	96.9
4	P (DM|Age ≥ 60, Family history = YES, Hyperlipidemia = YES)	57.0	78.9	78.9	97.6
5	P (DM|Age ≥ 60, Family history = YES, Hyperlipidemia = YES, Central obesity = YES)	57.0	81.0	81.0	98.0

*P(DM): It represents the original prevalence of DM in each dataset.

Furthermore, risk reasoning revealed that the BNs established by the three balanced datasets had a higher estimated probability of developing DM compared to the baseline value (the original prevalence of DM in each dataset) than the BNs constructed from the original unbalanced dataset, indicating that after balanced data processing, the performance of BNs in risk identification of DM was improved.

## Discussion

Diabetes is a metabolic disorder syndrome characterized by hyperglycemia, with an insidious onset and unobvious early symptoms. Although the control and prevention of non-communicable diseases such as DM have become a public health priority worldwide recently. However, data have shown steady increases in the number of people with DM in many countries^54–57^. It is estimated that 48% of the global population will be subject to DM by 2045, and the number of cases will rise to 629 million^58^. DM can lead to cardiovascular disease, kidney failure, diabetic foot and other complications, seriously affecting the quality of life of patients, imposing a serious economic burden on the families and society, and even leading to death in serious cases^59^. Therefore, it is of great clinical significance to analyze the risk predictors related to DM and study the network connection between them, which can help better carry out prevention, monitoring and self-management.

In this study, different BN models were developed based on the treated and untreated datasets to find out the risk factors of DM and then utilized them to predict the risk of DM. Boruta feature screening, widely used for data reduction in machine learning^31–33^, had been employed to select the 12 most significant predictors of DM before modelling, which can reduce the complexity of the network and avoid overfitting and misclassification. Afterwards, we compared the results of BNs based on three data processing methods (SMOTE, BL-SMOTE and SMOTE-ENN) and three BNs algorithms (Tabu, Hill-climbing and MMHC), and selected accuracy, sensitivity, specificity, PPV, NPV, AUC, MCC and G-mean values as the metrics to assess the classification performance of BNs models.

The results revealed that the BNs generated on the treated datasets by resampling can classify the DM better than that generated on the original imbalanced data. Moreover, the BNs combined with SMOTE-ENN and Boruta obtained the best performance of identifying people at high risk of DM from normal samples; and finding more risk factors directly associated with DM, compared to the BNs combined with SMOTE or BL-SMOTE resampling. Blind oversampling by SMOTE may result in generating too many unnecessary samples, which tends to break the class boundary and intensify the learning task^60^. Regarding BL-SMOTE, the KNN-based standard might fail to identify the marginal minority samples in some complicated data scenarios, where the synthetic minority samples may be incorrectly located deep within the region of other classes, thus making the learning task difficult^60^. It helps explain why the BNs combined with SMOTE or BL-SMOTE resampling do not perform as well as the BNs combined with SMOTE-ENN in this study. SMOTE-ENN, an integrated resampling method, first uses SMOTE to synthesize samples in the minority class samples and then combined with ENN to delete noise samples in the majority class samples^44,61^, so as to effectively make up for the drawbacks of SMOTE in producing noise samples and boundary samples^62^.

Among the three algorithms for constructing BNs structure models, Tabu exhibits superior classification performance. Tabu algorithm is an effective global optimization technique that combines adaptive memory to surpass local search and find the global optimum^63^. This method avoids repeating the same solutions by maintaining a mechanism called the "tabu list" and activates promising solutions using aspiration criteria. It has achieved favorable performance in many studies. Hill-climbing algorithm is a local optimization method that cannot guarantee finding the global minimum^52^. MMHC algorithm, as a classical hybrid algorithm, first utilizes the constraint-based MMPC method to infer the skeleton of BNs and then employs Bayesian scoring hill-climbing search to determine the direction of edges in the skeleton. However, hill-climbing is a local optimal algorithm, and the MMPC algorithm involves a high number of independence tests in the first-stage conditional independence testing, which can lead to inaccurate results^64^. These factors may contribute to the comparatively inferior performance of these two algorithms in this study, compared to the Tabu algorithm.

Therefore, after combining Boruta and SMOTE-ENN with the BNs constructed in Tabu Algorithm, seven variables strongly related to DM were detected. Utilizing the DAG, the complicated relationships between risk factors and DM were delineated clearly. Importantly, the interdependencies between these factors are in line with the biological and clinical interpretations. For instance, BNs reasoning shows that the risk of DM increases from 31 to 43.8% when an individual gets older or equal to 60 years old, which is consistent with the previous findings^65–68^. With the increase of age, the risk of developing DM keeps increasing, which may be due to the decreased pancreatic function in the elderly, leading to insufficient insulin secretion^69^. Additionally, the human body's demand for insulin may increase in some special circumstances, or the improper use of insulin in the elderly will lead to abnormal increases in blood glucose^70,71^. As DM is a genetic disease, one study has shown that the risk of developing DM doubles in people with a family history of DM compared with those without a genetic history^72^. As for BMI, the higher one individual's value is, the higher the fat content of the pancreas, which in turn affects the function of the pancreatic cells. Also, obesity may lead to insulin resistance, resulting in elevated blood glucose and increasing the risk of developing DM^73^. Dyslipidemia is related to the development of DM, and its lipid toxicity can affect the function of a *β* cell, increase the amount of free fatty acids, and enhance its oxidative metabolism in pancreatic *β* cells. Therefore, glucose metabolism is inhibited and insulin secretion is blocked, thus increasing the risk of DM^74^. Among all occupational groups, retirees were the most likely to develop diabetes, which may be related to the fact that most retirees are over 60 years old. Scholars^75^ have found that increased resting heart rate, one of the manifestations of autonomic nervous dysfunction, can cause an increase in sympathetic nervous system activity. Sympathetic activation may be the most important of many mechanisms leading to increased risk of DM^76^, causing vasoconstriction and reducing blood flow to skeletal muscle, leading to impaired skeletal muscle glucose uptake^77^. Additionally, it's associated with many DM-related risk factors, including decreased insulin sensitivity, obesity, high blood pressure, subclinical inflammation and metabolic syndrome^78,79^, all of which can increase the risk of developing DM. To make a more reliable conclusion, we took the significant factors shared by the three BNs models (constructed combining SMOTE-ENN, SMOTE and SVM-SMOTE techniques) as the most authentic determinants of DM, i.e., age, family history of diabetes, hyperlipidemia, central obesity (as shown in Tables 4 and 5).

In summary, the BNs constructed by combining Boruta and SMOTE-ENN not only found more risk factors directly related to DM, but also captured the probability relationship from the existing medical monitoring data by training data and self-learning. By separating direct and indirect dependencies, the potential unknown relationships of variables were revealed. Also, compared to the BNs built from extremely unbalanced raw data, or data balanced by SMOTE, BL-SMOTE, the BNs constructed by combining Boruta and SMOTE-ENN obtained the best classification performance (in Table 3). This combined approach could be a feasible method to detect the risk factors of DM and to demonstrate potentially complex network relationships between these factors.

Also, there are some shortcomings in this paper: (1) Our study lacked dietary-related factors because this characteristic was only present in the dietary survey households and the sample size was relatively small. In our ongoing work, we will consider combining multiple monitoring data to increase the sample size with this feature, and further incorporate it into the risk factors analysis of DM; (2) Given that this is a cross-sectional study, the causal relationship presented by the BNs needs further cohort studies verification; (3) The predictive performance of the warning factors found significantly related to DM needs to be further verified by external validation.

## Conclusion

A total of 4886 residents aged 18 and above were enrolled in our study, and 14.6% developed DM. Our study provided a simple, convenient, and effective combined model of Boruta, resampling and BNs to explore the relationships between demographics, lifestyle, physical condition and DM, as well as enabling for early detection of DM and research of factor network linkage effects, which will contribute to the prevention and control of DM, as well as the development of community work, facilitating the monitoring of DM patients and health management.

### Supplementary Information

Supplementary Tables.

## Data Availability

Data supporting the results of this study can be available by requesting the first author or corresponding author.
