# Supplementary material for: Diabetes mellitus early warning and factor analysis using ensemble Bayesian networks with SMOTE-ENN and Boruta
Source: Sci Rep. 2023 Aug 5;13:12718. doi: 10.1038/s41598-023-40036-5 (PMC10404250; doi:10.1038/s41598-023-40036-5)
Supplement: Supplementary file 1 — Supplementary Tables. [file 41598_2023_40036_MOESM1_ESM.pdf]

Diabetes mellitus early warning and factor analysis using ensemble Bayesian networks with SMOTE-ENN and Boruta

Xuchun Wang[a], Jiahui Ren[a], Hao Ren[a], Wenzhu Song[a], Yuchao Qiao[a], Ying Zhao[b], Liqin Linghu[a,b], Yu Cui[a], Zhiyang Zhao[a], Limin Chen[c,*], Lixia Qiu[a,*]

[a] Department of Health Statistics, School of Public Health, Shanxi Medical University, Taiyuan, Shanxi, China

[b] Shanxi Centre for Disease Control and Prevention, Taiyuan, Shanxi, 030012, China

[c] Shanxi Provincial People's Hospital, Taiyuan city, Shanxi Province, China

* Correspondence author

E-mail: sxchenlimin@163.com(LMC); qlx_1126@163.com(LXQ) ;

Univariate analysis results

Factors	Level	N	DM	Rate (%)	χ^2	*P*
Age	18~45	881	54	6.1	92.690	＜0.001
	45~60	2027	274	13.5		
	⩾60	1883	372	19.8		
Educational level	Elementary school and below	1989	332	16.7	13.544	0.001
	Junior and senior high school	2467	332	13.5		
	College degree and above	4791	700	14.6		
Marital status	Spinsterhood	151	12	7.9	9.684	0.008
	Married or cohabiting	4361	635	14.6		
	Divorced, widowed or separated	279	53	19.0		
Region	Rural	3193	442	13.8	4.525	0.033
	Urban	1598	258	16.1		
Occupation	Famers	2351	337	14.3	17.468	0.001
	Non agricultural	2003	273	13.6		
	Unemployers	147	24	16.3		
	Retirees	290	66	22.8		
Gender	Male	2153	315	14.6	0.001	0.972
	Female	2638	385	14.6		

Supplementary Table S1 Detection rate of DM with basic characteristics of different populations

Factors	Level	N	DM	Rate (%)	χ^2	*P*
Smoking	everyday	1184	154	13.0	5.848	0.119
	frequently	136	18	13.2		
	formerly	256	47	18.4		
	never	3215	481	15.0		
Drinking	currently	849	130	15.3	10.498	0.005
	formerly	362	32	8.8		
	never	3580	538	15.0		
Physical activity	Insufficient	3388	525	15.5	7.265	0.007
	Sufficient	1403	175	12.5		
Sleep (h/d)	＜5	578	108	18.7	9.012	0.011
	5~7	751	101	13.4		
	⩾7	3462	491	14.2		
Agrypnia	NO	2173	278	12.8	10.527	0.001
	YES	2618	422	16.1		
Mediation time (h/d)	＜3	1232	186	15.1	5.097	0.078
	3~5	1759	231	13.1		
	⩾5	1800	283	15.7		
Salt reduction	NO	2643	405	13.3	11.760	0.001
	YES	1743	295	16.9		

Supplementary Table S2 Comparison of detection rates and differences of DM in different lifestyles

Factors	Level	N	DM	Rate (%)	χ^2	*P*
Family history	NO	4137	508	12.3	143.302	＜0.001
	YES	532	168	31.6		
	unclear	122	24	19.7		
Central obesity	NO	1840	176	9.6	60.955	＜0.001
	YES	2951	524	17.8		
Hypertension	NO	2667	278	10.4	84.534	＜0.001
	YES	2124	422	19.9		
Hyperlipidemia	NO	1679	157	9.4	57.322	＜0.001
	YES	3112	543	17.4		
BMI (kg/m^2)	＜18.5	97	7	7.2	34.791	0.001
	18.5~24.0	1885	217	11.5		
	24.0~28.0	1923	309	16.1		
	⩾28.0	886	167	18.8		
Heart rate(times/min)	＜60	52	5	9.6	7.202	0.027
	60~100	2403	380	15.8		
	⩾100	162	37	22.8		
Hemorrhagic apoplexy	NO	4761	694	14.6	0.703	0.402
	YES	30	6	20.0		
Cervical and lumbar disease	NO	3657	3135	14.3	1.404	0.236
	YES	1134	178	15.7		
Chronic digestive diseases	NO	4142	609	14.7	0.209	0.648
	YES	649	91	14.0		
Anaphylactic disease	NO	4503	655	14.5	0.304	0.581
	YES	286	45	15.7		

Supplementary Table S3 Comparison of detection rates and differences of DM in different physical condition
